# Supplementary material for: Patterns of Intron Gain and Loss in Fungi
Source: PLoS Biol. 2004 Nov 30;2(12):e422. doi: 10.1371/journal.pbio.0020422 (PMC532390; doi:10.1371/journal.pbio.0020422)
Supplement: Table S1 — Also available at http://genes.mit.edu/NielsenEtAl/. (4.3 MB ZIP). [file pbio.0020422.st001.zip › NielsenEtAl/html/1368.html]

AN3019.1.NCU10067.1.MG03512.1.FG07938.1


```
 CLUSTAL W (1.82) Multiple Sequence Alignments - Introns Inserted


Sequence 1: NCU10067.1	289 aa
Sequence 2: MG03512.1	291 aa
Sequence 3: FG07938.1	270 aa
Sequence 4: AN3019.1	297 aa
Alignment Length: 328 aa
Number Identitical Residues: 115 aa
Alignment Score (without introns) 5650


MG03512.1 	----MAER------------~----QLQQILTQLKS--QQLAYADAAKLLSKAKLLLLQL
NCU10067.1	----MAER------------~----QLTQILTQLQTSASSLSYTDSSALLSKAKLLLLQL
FG07938.1 	----MAER------------~----NLSQIISQLKHS-PSMSYTDANALLSKAKLALLKL
AN3019.1  	MVADMSPRDLTAKPCGEAVS2AMASELRSLVGDLTNAVNRKQFDTANTLLARAKRTLLLQ
          	  :.*: *. ::.....: : : :::* .:: :*  :     :  :  **::**  **  

MG03512.1 	GALTPKTTTTTSSSTSSGKGKSSSSGGASPSAAQLALARETYEQGALYSIRAKNADGFTR
NCU10067.1	NALTP-----------------TAPGSAS-NPSLFSLARQVYEQGALASIRAKNPDAFLR
FG07938.1 	NALTP---------------------SPSTPTQLLPLARETYEQGALFAIRARNPEAFTR
AN3019.1  	NALIP---------------------ASSTNPELLVLARETLELGALSAIRQTDAPAFTR
          	.** *                     ..*. .  : ***:. * *** :**  :. .* *

MG03512.1 	YVQQLSPLYELPANVLP-----------PNLPERNKVTGLYLLLLLTQGRYAEFHSELEA
NCU10067.1	YVSQLQGFYELESTVGTGAAGGRIAGADANREEKNKVTGLYLLLLLTQGQYAEFHSELEA
FG07938.1 	YVQQLQPFYELPASVLP-----------PNLLERNKVTGLSLLLLLTQGRYAEFHTELES
AN3019.1  	YYQQLQPFYDLEREGSS--------TLDTRTSQRSKITGLYLLLLLSSGDGSSFHTVLEG
          	* .**. :*:*     .          ...  ::.*:*** *****:.*  :.**: **.

MG03512.1 	LSTREGGGGSVDVEGDRYLGYPIRLERWLMEGSYDRVWKAMKKGEVPCEEYGVFSE0ILT
NCU10067.1	LSTRLGG-ATRELEADRYLGYPIKLERWLMEGSYDRVWKAMKKGEVPCEEYGVFSE0ILT
FG07938.1 	LENREGG--GGDVESDRYLGYPIRLERWLMEGSYDRVWKAMKSSEVPCDEYSVFSE0ILK
AN3019.1  	L-VEEASLKGGNVEDDPFIKYPVNLERNLMEGSYDKVWRETNSERVPSEDFALFSN0VLV
          	*  . ..    ::* * :: **:.*** *******:**:  :. .**.:::.:**: :* 

MG03512.1 	IQIRAEIASSSERAYPSLPISSTKSLLFLESEGAVVEFAKRRGWTVRDGHIYFPTAG--A
NCU10067.1	FQIRSEIASSSERAYPSLPISSTKSLLFLESEGEVVEFARSRGWVLKDGHIYFPSAEPTT
FG07938.1 	NQIRSEIASSSERAYPSLPISSTKSLLFLDSEGDVISFAHHRGWIVRDGHIYFPDAAE-G
AN3019.1  	GTIRSEIADCSEKAYPSLPISNAKNLLFLESEGAVMEFAQQRGWTLRDGRIYFPVEPEAA
          	  **:***..**:********.:*.****:*** *:.**: *** ::**:****    : 

MG03512.1 	EDG--EPEKPVSLSFIENTLGYARELETIV
NCU10067.1	EEAGAEQEKPFSRLIIENTLGYARELETIV
FG07938.1 	ENG--DQNKEMSQMVIENALGYARELETIV
AN3019.1  	TRS-EKDILVASSTIIENAIGYARELETIV
          	  .  .     *  .***::**********
```
